# Osteoblastic bone metastases from renal cell carcinoma

**DOI:** 10.2478/raon-2013-0034

**Published:** 2014-07-10

**Authors:** Vladka Salapura, Irena Zupan, Bostjan Seruga, Gorana Gasljevic, Pavel Kavcic

**Affiliations:** 1 Clinical Radiology Institute, UMC Ljubljana, Ljubljana, Slovenia; 2 Clinical Department of Haematology, UMC Ljubljana, Ljubljana, Slovenia; 3 Sector of Medical Oncology, Institute of Oncology Ljubljana, Ljubljana, Slovenia; 4 Department of Pathology, Institute of Oncology Ljubljana, Ljubljana, Slovenia

**Keywords:** renal cell carcinoma, osteoblastic bone metastases

## Abstract

**Background:**

RCC accounts for only 2–3% of all cancers. Due to its’ non-specific symptoms disease is often diagnosed in advanced stage. Disseminated RCC frequently produces bone metastases that are almost always highly destructive, hyper vascularized and purely osteolytic.

**Case report.:**

In this article we describe a case of a 71-year old male patient with disseminated osteoblastic bone metastases from renal cell carcinoma (RCC), and present a short review of published literature reporting cases of osteoblastic bone metastases from RCC. Our patient presented with thoracic pain aggravated by movement. He was diagnosed with predominantly osteoblastic bone metastases in the skeleton of thoracic and lumbar vertebra along with metastases in iliac bones, ribs, humerus and clavicles. Initially, origin of bone metastases was unknown, but later a small tumor in patient’s right kidney was identified. Microscopic evaluation of the open bone biopsy showed clear cell RCC with sarcomatoid differentiation.

**Conclusions:**

Although, due to its’ rarity, RCC is not included in the primary differential diagnosis in patients with osteoblastic metastases, such rare cases suggest that RCC may be considered in the diagnosis when there no other primary tumor is found.

## Introduction

Renal cell carcinoma (RCC) accounts for 2–3% of all cancers.^1^ Due to non-specific symptoms disease is often diagnosed relatively late. Approximately one third of patients with newly diagnosed RCC already have metastatic disease.^2^ Metastases to the bones are frequent and occur in 35% to 40% of cases with advanced RCCs.^3^ Usually, these metastases are highly destructive, hypervascular and osteolytic.^3^ In a series of 1668 patients with RCC all detected bone metastases were exclusively osteolytic prior to the initiation of therapy.^4^ It is well known that specific therapies, such as radiotherapy may induce sclerotic changes in osteolytic bone lesions.

To our knowledge there are only six cases of patients with osteoblastic metastases from RCC reported in the literature: two involving well differentiated RCC^5^,^6^; two involving RCC with sarcomatoid differentiation^7^,^8^; one mixed clear cell with oncocytic features^8^ and one unclassified type.^8^ In this article we represent a case of osteoblastic bone metastases from clear cell RCC with sarcomatoid differentiation.

## Case report

**FIGURE 1.**
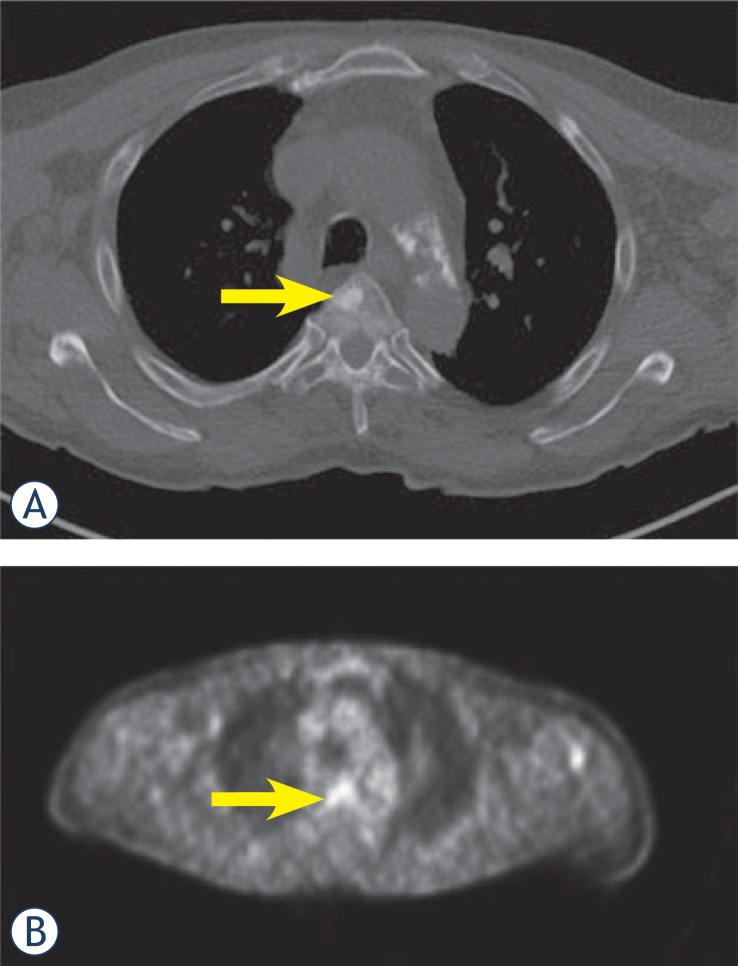
A. CT scan shows osteoblastic metastasis in the anterior part of thoracic vertebral body (arrow); B. PET-scan shows that the lesion is metabolically active (arrow).

**FIGURE 2.**
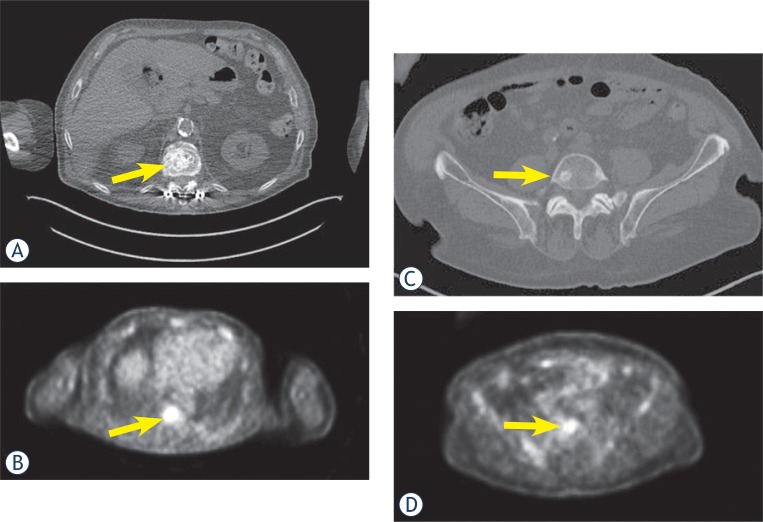
A and C. CT scan shows osteoblastic metastases in lumbar vertebral bodies (arrows); B and D. the same lesions show metabolic activity on PET-scan (arrows).

**FIGURE 3.**
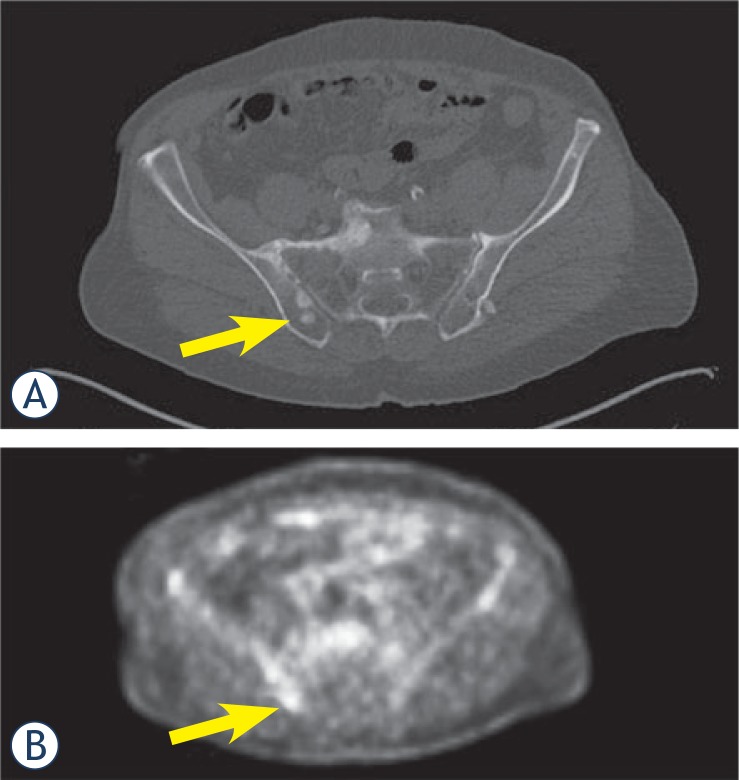
A. CT scan shows multiple osteoblastic metastases in the right iliac bone (arrow); B. PET-scan confirms these lesions to be metabolically active metastatic lesions (arrow).

**FIGURE 4.**
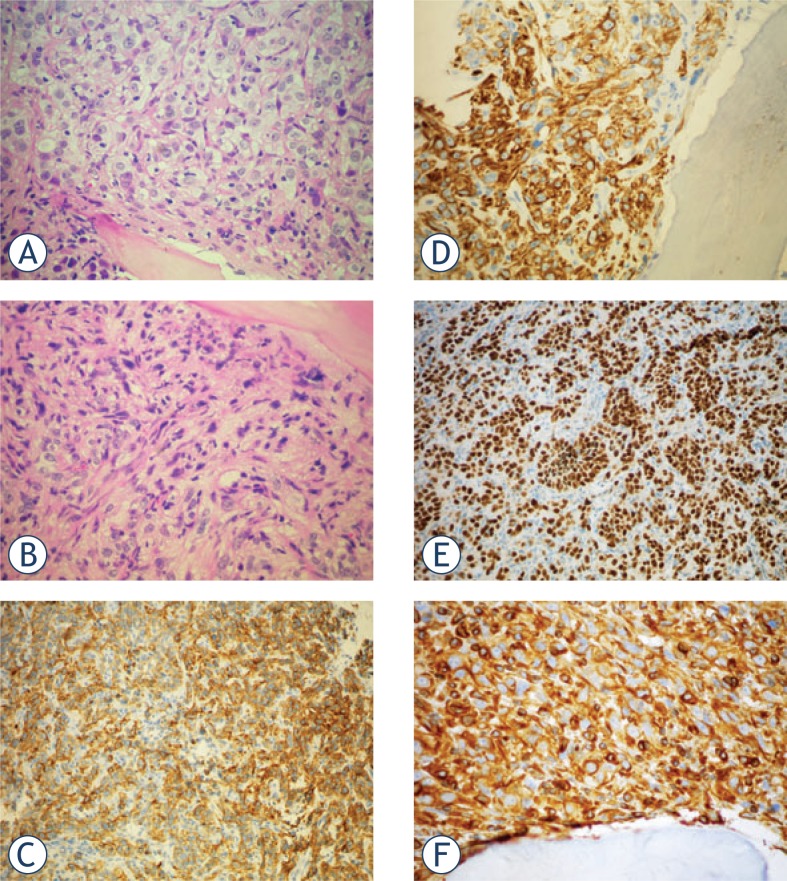
A. Metastasis of the RCC in the bone: cells with copious clear cytoplasm and nuclei with prominent, eosinophilic nucleoli; bone trabecule is in the bottom part of the field; H&E 40x; B. More spindled tumor cells, »sacomatoid« differentiation; H&E, 40x; C. Positivity for CAM5.2; IHC CAM5.2, 20x; D. Positivity for RCC; IHC RCC, 40x; E. Positivity for PAX8, IHC PAX8, 20x; F. Positivity for Vimentin; IHC Vimentin, 40x.

71-year old male was admitted to emergency department due to a chest pain that was aggravated by moving or breathing. Patient also had unexplained weight loss of 22 kg in the last 3 months. Medical history included arterial hypertension, diabetes and chronic kidney disease. Laboratory data showed normocytic anemia, elevated inflammatory parameters, elevated alkaline phosphatase and creatinine. Chest X-ray did not show any abnormalities. Initially, pulmonary embolisms were suspected, but chest computer tomography angiography (CTA) did not show any abnormalities in the lungs. Abdominal ultrasound examination found already known adrenal adenoma with no other abnormalities. As attending physician was suspicious of malignant disease patient was hospitalized. During hospitalization 18F-fluorodeoxyglucose PET/CT imaging was done which revealed disseminated predominantly osteoblastic metastatic lesions in thoracic (Figure 1) and lumbar spine (Figure 2), iliac bones (Figure 3), ribs, humerus and clavicles. However, primary tumor was not identified initially. For further characterization magnetic resonance imaging (MRI) was planned, but unfortunately it was contraindicated due to more than 20 years old osteosynthetic material present in the lumbar vertebra. A CT guided biopsy of small osteoblastic lesion in the iliac crest was ordered. Unfortunately, biopsy was inconclusive, since it showed only a fatty bone marrow without any malignant cells. Finally, patient underwent an open bone biopsy of large osteoblastic lesion in the eleventh thoracic vertebra. Microscopic evaluation of the open biopsy showed clear cell RCC with sarcomatoid differentiation (Figure 4).

**FIGURE 5.**
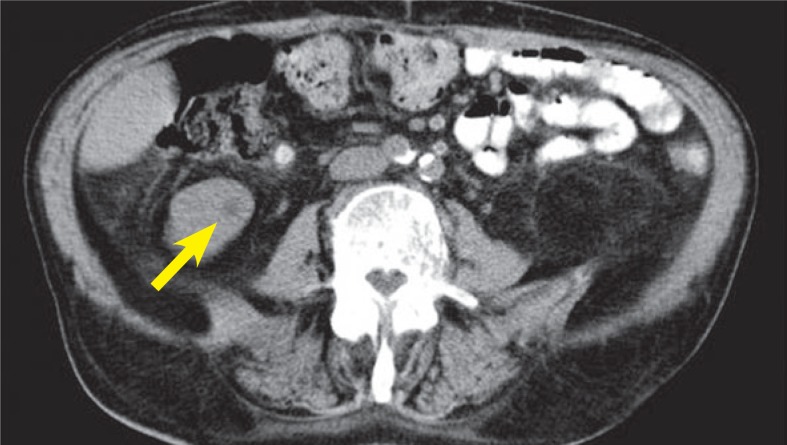
Abdominal CT scan shows small primary renal tumor in the the right kidney (contrast enchanced CT was not performed due to patient’s poor renal function with glomerular filtration less then 30 mL/min/1.73 m^2^).

Afterwards patient was presented to medical oncologist. Control CT scan confirmed a small carcinoma in the right kidney (Figure 5) with diffuse bone metastases, which were predominantly osteoblastic. Additionally, numerous new tiny lung metastases were found leaving no suspicion that primary tumor was not in the lungs. His prostate specific antigen was 0.3 ng/ml and therefore it was very unlikely that osteoblastic metastases were from prostate cancer. At presentation patient had several poor-prognosis risk factors (WHO performance status 2–3, anemia and time from initial diagnosis to the start of treatment less than 1 year), which indicated short life expectancy. He was offered treatment with mammalian target of rapamycic (mTOR) inhibitor temsirolimus. Unfortunately, despite treatment with temsirolimus patient gradually deteriorated and after two months of treatment CT scan showed progression of disease in his lungs. Few weeks later patient died.

## Discussion

The most common cancer types that metastasize to the bones are prostate, breast, lung cancer and RCC.^9^ When osteoblastic bone metastases are found in an adult male patient the most likely origin of malignancy is prostate cancer. However, when osteoblastic bone metastases are found in conjunction with an enhancing renal mass, the more likely pathology is urothelial carcinoma. Urothelial carcinoma such as transitional cell carcinoma commonly metastasizes to bones and can produce both osteolytic and osteoblastic metastases.^10^ RCC almost always produces osteolytic metastases and is therefore usually not considered in the differential diagnosis of osteoblastic metastases. However, advanced RCC can present with osteoblastic metastases as was also found in our case.

RCC is made up of a number of different histological subtypes and each is caused by alterations of different genes. The common sites of metastases are lung, liver, bones, adrenals and lymph nodes.^11^ Some RCCs are associated with unfavorable histological features such as sarcomatoid differentiation, which indicate aggressive behavior. Our and two previously published case reports^8^,^9^ of patients with osteoblastic bone metastases from RCC reported sarcomatoid differentiation in bone metastases. This shows that sarcomatoid differentiation in bone metastases from RCC can be associated with osteoblastic metastases.

Patients with metastatic RCC of bone have expected median survival of around 12 months; survival of those with sarcomatoid differentiation can be even worse.^12^,^13^ Now, the outlook is changing thanks to the advancements in targeted molecular therapy and cytoreductive nephrectomy.^11^ RCC is an immunogenic tumor that has ability to manipulate and suppress the natural immune system. The primary tumor might suppress the antitumor effect of the host defense mechanism and divert the immune cells away from the distant metastases.^14^ Hence, removal of primary tumor (cytoreductive nephrectomy) together with additional immunotherapy can augment the host immune system thereby producing better survival and quality of life.^11^

New imaging techniques are also being developed. A recent study presented the first clinical validation of a molecular imaging biomarker for malignancy. It was shown that highly malignant clear cell RCC can be identified using (124) I-girentuximab PET/CT imaging with high sensitivity and specificity.^15^

With the progression in diagnostics, surgery, new radiotherapy techniques and the discovery of the new biological therapies which are more effective and less toxic, major changes of the therapeutic results are expected. In spite of this, RCC still remains a big challenge for the future research.^16^

## Conclusions

In this article we presented a rare case of RCC with predominantly osteoblastic metastases. Although, due to its’ rarity, RCC is not included in the primary differential diagnosis in patients with osteoblastic metastases, such rare cases suggest that RCC may be considered in the diagnosis when there no other primary tumor is found. Especially, as survival of patients with metastatic RCC continues to increase due to new therapies, we may begin to see unusual radiologic characteristics of metastases, (such as osteoblastic metastases) more often. Ongoing research in treatment and imaging will help to optimize management of metastatic RCC in the future.
